# Remarks on Hydrophobia

**Published:** 1809-07

**Authors:** Louis Valentin

**Affiliations:** M.D.


We have at last been able to renew our Correspondence with the Continent, but we fear in an interrupted form. The following Article is selected from the JOURNAL DE MEDICINE, on account of the experiments with which it concludes.

*Remarks on Hydrophobia, contained in a Letter addressed by* LOUIS VALENTIN, *of Marseilles, M.D. to* ALEXANDER HALDAT, M. D. *Secretary to the Academy of Nancy.*

HYDROPHOBIA presents so vast and extensive a field for observation, that I must content myself with a mere outline of the subject, in order to introduce some facts which I formerly collected at Nancy, and which must be fresh in the recollection of many of the inhabitants.

It is clearly ascertained, that after the bite of a mad animal, the local treatment by actual cautery is generally the best that can be resorted to.

In vain have an infinite number of external and internal applications been tried; all of them have had their advocates, and almost all have been attended with success, real or imaginary, but time and experience have done justice to most of them. Medical science however is still in want of a remedy aginst this most formidable malady. It is painful to reflect that not a single curative remedy has been applied to it. Amputation, excision, and cauterising are the only means which present a probability of prevention.

Skilful practitioners agree that the rabid virus, when deposited posited in the wound, is not destroyed unless it be instantly and properly cauterized, in order to prevent the absorption of the poison by the lymphatic vessels, and its subsequent transmission into the circulation. It is perhaps more hypothetical to believe in the mixture of the poisonous saliva with the mass of fluids, in order to infect them by circulating throughout the whole body for weeks, months and years, than to deny it entirely. It occurs to me, however, that this question is still undetermined.

May we not suppose that the first effects of this poison *sui generis*, only pass along the nervous fibres, with which it remains in contact or in a state of fixity, and that it is only in virtue of the *consensus* that the disease is subsequently transmitted throughout the whole machine, after an indeterminate time producing its symptoms?* That a sufficient number of accurate observations may resolve this question, I should propose, in order more speedily to obtain its solution, that the local treatment be resorted to at all stages of the disease. That is to say, if the operation has not been performed immediately after the accident, that it should be attempted at subsequent periods, either on the first appearance of symptoms, or when hydrophobia has come on, without neglecting, however, the antispasmodic remedies.

Within a short time, M. Rigal, surgeon at Gaillac department of the Tarn, has preserved the lives of four persons, by cauterizing them nine days after the bite of a mad dog.

Dr. Gallup, of Woodstock, in Vermont, North America, informed Professor Mitchell, in July 1806, that having been called in to a man, 28 years of age, who twenty-five days before had been bitten three inches above the left hand, the Doctor found the cicatrix soft, and at times painful: he cut out the cicatrix by a circular incision, applied caustic alkali to the wound, and dressed it with blistering plaister. He gave mercurials internally for two months. Several dogs, two pigs, and a sheep, which were bitten on the same day with this man, and by the same animal, died rabid. When Dr. Gallup drew up the above account his patient had remained well fifteen months. Success is always less doubtful before the appearance of spasms and of hydrophobia; but when the danger is imminent, why should we

* *Roux*, of Dijon, in common with many others, is of this opinion.

we not act with boldness, and resort to the most desperate remedies?

The application of red-hot iron should almost always be preferred to caustics; indeed, the latter ought to be entirely excluded in the last stage of the disease. Where the bite is deep, if the cicatrix be visible, some incisions should be made before the place is cauterized; for in all cases it is of the utmost importance to destroy every thing that may have been infected, and to excite speedily in the part an inflammatory stimulus capable of exalting the vital energy, and afterwards producing an abundant suppuration. The want of this precaution has sometimes been the cause of failure in the operation, and in every local application.*

The poisonous molecules in the animal's saliva, introduced into the skin or cellular substance, do not display their effects upon the system, nor produce the convulsive agitations until a period more or less remote. The term of forty days, more or less, has generally been fixed: several are seized with hydrophobia in twenty or thirty days. The greatest number, however, are seized after this time, and some upwards of a year from the period of the bite. In general, however, the interval between the bite and the first symptoms does not exceed five or six months. If it be true that these deleterious molecules remain stagnated, and as it were dormant in the bitten part, and do not sympathetically exhibit their effects upon the nervous system until after a certain time of incubation,† we ought to follow the same practice as in *Tetanus*.

The

---

* Vide Enaux and Chaussier, Traitè sur le Rage. In some instructions for the prevention of Hydrophobia, published by order of the Intendant of the Police of Paris, in 1785; it is directed, when madness has come on, to apply the actual cautery, or the moxa to the lower extremities, and cupping glasses to the shoulders.

† What is called the *local operation of other fixed viruses*, is also more or less tardy, and sometimes depends upon unknown circumstances which do not seem embarrassing to those who think they can explain every thing. Altho' the insertion of the variolus and vaccine virus produces their effects after a term nearly regular, there are several instances of deviation from the general rule. Vaccination presents these anomalies most frequently. The most remarkable known in England, is related by Mr. Ring, the friend of Dr. Jenner. In a patient whom he had vaccinated, the pustule was only developed on the fortieth day, and the test of small-pox inoculation, ascertained that the child had been vaccinated.

But on the Continent a more extraordinary case occurred, perhaps unique in the annals of vaccination. My old friend, Dr. Clerc of Metz, informed me, that M. Chevreuse, an apothecary of that place, last year vaccinated a child four or five years old in two places on each arm, and that it was a whole year before the vaccine infection completely shewed itself Dr. Clerc saw the virus going through its different stages, and noticed the wounds

The same course ought to be followed with the bites of venomous serpents, notwithstanding the extreme energy of the poison and the promptitude of its action. Although contrary opinions have certainly been given, it appears to me from what I have seen in America, that surgical operations, by incision and burning, succeed more efficaciously than any internal remedies; the application of plantains, the root of *polygala seneka,* and oily, alkaline, or ammoniacal embrocations. I must admit however, that all these, as topical remedies, justly hold the second rank.

I have had opportunities of seeing many persons who were bitten by mad animals; and I have seen several die of hydrophobia, notwithstanding the best treatment as prescribed by modern authors.* At Nancy I had three patients of this description under my care; two were cured, the third had not been cauterized. One of these persons consulted me a few days after he had been bitten in the leg; I applied the actual cautery to the wound, and I recommended it to him to keep up the suppuration. This man took no medicines except some doses of ammonia in an infusion of flowers of the linden tree, and no accident whatever happened.

M. de la Lawrencie Neuvich, major in the King's regiment of infantry, kept two greyhounds. Towards the end of 1783, one of them bit him very violently in the middle of the forepart of the thigh, making a wound of about an inch long. The late M. Deyoteux and myself attended him with the utmost care. We cauterized the wound immediately after the accident, and prescribed a complete course of mercurial frictions, which did not produce salivation. The patient also took some attempering antispasmodic remedies, and observed a proper regimen. Being naturally endowed with great sensibility, he was confined upwards of two months to his room, where he used to read medical books. He suffered no subsequent inconvenience from the bite, and he is still alive.

As it was very important to ascertain if the dog which bit him was really affected, he was shut up by himself, and he died mad. The other dog, which had been also bitten, was treated in the same way, and he exhibited the same fatal symptoms.

In

* The celebrated M. Petit, ex-surgeon to the Grand Hospital of Lyons, says, that he never saw a patient recover after hydrophobia had shewn itself.

In 1787, three years and a half after this event, a child of ten years of age, named Sigisbert Viriot, living in the Faubourg St. Pierre, was dreadfully bitten by a strange dog, which left twelve or thirteen wounds, four or five on the left hand, and a very deep one in the left leg. One of his elder brothers, Francis Viriot, forced the dog from the child and killed it. At the same time he was himself bitten on the back of the right hand, but told no person, nor did he take any precaution. As it was not very certain if the dog was mad, a piece of meat was rubbed upon its throat, in order to impregnate it with saliva, and it was given to other dogs, but they refused to eat it. This refusal of a healthy animal, has been regarded by some as a test of hydrophobia having existed in the dead animal, but modern writers think it is illusory.

On the same day, and a very short time after this accident, incisions were made into the bitten parts; they were allowed to bleed freely, and were afterwards washed with a solution of common salt. Care was taken to keep up the suppuration a long time, and to sprinkle the sore sometimes with cantharides mixed with mercurials, or digestives, which served as dressings. Being at that time the family physician, I was entrusted with the care of this child, and employed mercurial friction, until the salivary glands were slightly affected, and kept them in this state. Mercury was also rubbed in around the wounds, the largest of which were kept in a state of suppuration for five or six weeks.

Upwards of fifty days after the accident, the course of medicine being finished, and the child in appearance quite well, he obtained leave from his parents to spend a holiday at St. Nicholas with his two brothers, one of whom had been bitten in the hand, as above mentioned. He returned home in the evening on foot, and in the midst of a heavy rain, with his clothes wet through. He immediately complained of great thirst, and called for water, which his mother gave him. At the sight of it, he shivered, his throat seemed shut, and he could not swallow it: his whole body trembled, and he passed the night in a state of spasm and continual agitation. Next day Professor Tournay and myself were called in. In vain did we attempt to make him swallow any liquid. He begged that it might be taken away from him: the slightest agitation of the air, or the sight of a fluid threw him into convulsions. All was over with him, and the hydrophobia was at its height. He took some opium pills however, and Dr.

Tournay

Tournay prescribed an omulet, into which calcined oyster shells were introduced. I do not recollect if he took it; but he died on the third day in convulsions.

The brother, who had been bitten by the same dog was not alarmed. He continued to conceal the circumstance, and no bad consequence ensued. He only communicated it to me in 1807, (twenty years after the bite) at Marseilles, where I met him accidentally. I saw the cicatrix, which was upwards of half an inch long, situated transversely upon the back of the right hand, above the space formed inferiorly by the last two metacarpal bones. This gentleman (Francis Viriot) is at present a lieutenant colonel and aide-de-camp to the prince of Yssembourg.—We may be permitted perhaps to suppose, that this favorable case can only be ascribed to the defect of the insertion of the rabid virus into the wound, from the slaver of the animal having been expended upon the other child, and perhaps absorbed by the stocking which covered his leg.

I have made many other attempts at a preservative treatment, but I was not always convinced that the animal which inflicted the wound was really mad. It is of the greatest importance to avoid if possible killing the suspected animal immediately; it should be shut up and carefully observed, when the event will regulate the conduct of the physician. If the animal is not mad, the person bitten will be at ease.

Canine madness is extremely rare in the warm regions of South America. I was assured at St. Domingo, in 1791, that it was formerly unknown in the Antilles, and that it was only within these thirty or forty years that it was seen there. A man and woman died at Cape Français when I was there, from being bitten by a cat, which was not suspected. Who has not heard of the ravages made by a dog in the Limonade upon several negroes who died of hydrophobia? An intrepid negro, named Coucouba, seeing this furious animal enter a house, armed himself with a knife, marched up to it naked, and attacked it. He was not victorious, however, until he had received several wounds on various parts of his body. Incisions were made; gunpowder was fired upon the wounds, and mercurials were resorted to. This treatment succeeded, and Coucouba never displayed any symptom of hydrophobia.*

Canine

* Vide Journal de Med. tome lxx. p. 531.

Canine madness is common in North America, where they have not been more successful in its treatment than in Europe. During a long time in Connecticut the prompt application of common salt to the bite was thought to be a sufficient preventive. The savages, with more certainty, annihilated the contagious matter by applying fire to the bitten part, on which they burnt, in preference to any other wood, the bark of the white ash. This process, which radically extirpates the evil, was not taught them by any stranger.

Negroes in the United States, and particularly in Maryland, have suffered no inconvenience from eating pigs which died rabid. A whole family drank the milk of a cow seized with hydrophobia the same day, without any detriment to their health. These facts confirm those related by Dr. Baumgarten in the Medical Commentaries, and by several European authors, who attest their having used with impunity the flesh and milk of rabid animals.

The celebrated Dr. Rush, of Philadelphia, compares hydrophobia to a malignant fever in its highest stage. He founds his opinion on the progress, the symptoms of the disease, and the state in which the organs are found on dissection. Besides the local treatment and a proper regimen, the basis of his plan consists in repeated bleedings, and in the use of mercury. In most cases, according to him, from one to two hundred ounces of blood should be taken from the patient.*

I shall take this opportunity of detailing the process suggested by another physician of Pennsylvania. Doctor Phisick, of New York, persuaded that the death of patients of this description is occasioned by the spasmodic constriction of the glottis, hence producing gradual or sudden suffocation, has proposed to practice *Laryngotomy*. By this operation, (prescribed in Europe for inflammatory and suffocating angina, and rarely practised) he thinks that in facilitating the admission of air into the lungs, by means of an artificial passage, there would be a greater probability of a cure, in consequence of thus gaining time for the exhibition of remedies. He thinks, that the aversion to water, proceeds from the convulsive contraction of the muscles of the glottis, hindering the patient from breathing, and threatening suffocation every time that he attempts to swallow liquids. Dr. Phisick

* Observations upon the Nature and Cure of Hydrophobia.

sick hazarded this opinion in 1801, upon inspecting the body of a negro who died of hydrophobia, and in whom, however, he found the larynx and the tracheal artery in the natural state. (Medical Respository, vol. v.)

In 1802, the same physician was on the point of performing this operation upon a child five years old, attacked with hydrophobia, in consequence of the bite of a mad dog. The breathing and cough were similar to tracheal angina, or the croup, in a moderate degree. While he was preparing the instruments, in the presence of Drs. Rush and Griffiths, the child expired. Dr. Rush, who opened the body, found the glottis and epiglottis inflamed. The glottis was so thickened and close, that it scarcely admitted a small probe. Below, the trachea was similarly inflamed, thickened, and contained mucus, as has been frequently observed in patients who died of croup.

It is not my object to bring under view the great number of remedies which have been employed in hydrophobia. Some having more particularly fixed the attention of the public, have obtained a certain degree of reputation; it may therefore, be necessary to ascertain their value, particularly the more recent, by further experiments. Notwithstanding the former reputation of *belladonna* and the *anagallis arvensis*, they have now fallen into disrepute, and it will probably be the same with the bark of the Mico coulier, (Celtis australis) the virtues of which have been so much spoken of. Mercury has retained the most partizans, and it seems to be superior to other remedies, particularly if combined with antispasmodics. How often, however, has it been inefficacious when the bitten part was not cauterized? The case of young Viriot and a thousand others are proofs of this.

Pills of cantharides and cinnamon, with the warm bath, have been used with advantage in Austria. Werlhoff bestows great praise on one grain of cantharides, one grain of sweet mercury, and twelve grains of camphor, made into a bolus with gum tragacanth, to be taken every night for six weeks. But he employed burning and mercurial frictions at the same time.

Dr. Fothergill, who published seven or eight years ago a work "On the nature of the disease occasioned by the bite of a mad dog," in which he considers hydrophobia as a kind of spasmodic angina, thinks also that the best method of breaking the fatal connection between the diseased part and the organ of deglutition is cautery. In the the violence of the spasms, when the disease is declared, he advises the whole body to be rubbed before a gentle fire with a pound of olive oil, in which half an ounce of camphor has been dissolved. He recommends also that the patient should be immersed in a bath of warm olive oil, if it can be procured, in order to excite an abundant perspiration, and that a blister should be applied to the neck from ear to ear.

Two substances, diametrically opposite in their nature, the acids and the alkalis, have been recommended. In Prussia, Reich boasts of the mineral acids. In France, Lassonne, Sage, and others, have again called the attention of the profession to ammonia. Boerhaave had already prescribed vinegar; and Clerc, in his Natural History of the sick Man, gives an instance of a cure obtained with five ounces of this acid, taken thrice a day. Half a bottle swallowed at a draught, and by mere accident, cured a patient in Italy. Nothing more was wanting to confirm the reputation of vinegar. This calls to my recollection a circumstance which happened eleven years ago at Chaumerac, near Privas, in the department of Ardeche, which was attested to me by an eye witness, now living at Marseilles.

A very large pig was attacked with hydrophobia in consequence of being bitten by a mad dog. A countryman was allowed to undertake the cure, upon assuring the owner that he had experienced the success of the method he was about to try. The animal was shut up in a stable, where it began furiously to gnaw pieces of wood, or any hard substance that was thrown to it. The countryman made a large hole in the roof of the stable, through which he let down a saucepan containing about two pails full of warm vinegar. The animal instantly ran to the liquid, and drank every drop of it. In a few minutes the madness ceased, and the animal was cured.

The application of viper venom to men and animals already bitten by a mad dog, has not met with the success which two or three examples had given reason to hope. According to Dr. Desgranges, of Lyons, a large mad dog, bitten by a viper, died with all the usual symptoms of hydrophobia. Two persons, upon whom a similar experiment was tried at the Hospital of Lyons, also died in the same way.

M. Gauchi, mayor of Reorthe, has lately announced, the best method of preserving animals, and particularly dogs, from hydrophobia, is to have them bitten by a viper previously previously to their being bit by the animal which is rabid; because, according to his opinion, the poison of the viper does not neutralize the poison of the hydrophobia, if applied after the bite of the dog. His experience taught him, that dogs which have been bitten by rabid animals, did not become rabid if previously bitten by a viper, and a swelling had been produced. (Journal d'Economie Rurale, 1805.) If the good effects of this process were well authenticated, it would prove a new kind of inoculation, which would have the same salutary influence on animals that vaccination has on mankind, as a preservative against small-pox.

In a memoir read by Professor Anselmi to the Imperial Academy of Turin, we find the case of a man cured of hydrophobia by galvanism. This remedy was applied during the first symptoms of hydrophobia, and the individual who was cured, was presented to the academy at the sitting at which the memoir was read.

Some authors, and latterly one of our colleagues in the academy of Nancy,* admitting spontaneous hydrophobia, deny that this disease is ever exhibited in persons who have been bit by animals not actually rabid. There are irrefragable proofs, however, of persons who have died mad after being bit by dogs and cats who were not at all affected. Besides, the case mentioned by Theodore Swinger, the case of Lister, inserted by Bonnet in his *Sepulchretum*, is quoted as one of the most remarkable.

Professor Rossi published in the memoirs of the Turin academy for 1801 and 1802, three cases of persons who died of hydrophobia, after being bit by cats which were not mad, but which had been greatly irritated previous to the bite. In these cases the axiom, *Nemo dat quod non habet*, does not apply. It is remarkable, that the third case was that of a man who attacked a cat with a small hatchet in a dairy. The animal seeing no possibility of escaping the mortal blow, flew upon the man and seized his chin so forcibly, that it could not be detached until the head was cut off. The patient was carried to the hospital, where he was cauterized, bled, purged, and underwent a mercurial course. On the 20th day, however he became hydrophobic, and afterwards so furious that he broke his chains,

* Dr. Gorey—"Reflexions sur le Rage, &c. Journ. de Med." de Corvisart et de Roux, February 1807.

chains, ran out, and fell down dead at the gate of the hospital.

This confirms the opinion of several physicians, who thought that the saliva of an animal exasperated to extreme fury contracts a particular quality, and a morbid alteration, which renders it capable of communicating the disease. Some even furnish examples of epileptic and feverish patients, whose bite produced this disease. Cases of this description are however very rare.

In 1789, M. de Beausett, captain in the King's regiment of infantry, at Nancy, being in a violent paroxysm of epilepsy, seized with his teeth the point of my fore-finger while I was in the act of introducing some salt into his mouth; he pressed it between his teeth with such force for two or three seconds, that I thought he had divided it. I found I had received two contused wounds precisely upon the sides of the articulation of the last phalanx, which was covered with frothy saliva. Notwithstanding the fears of the bye-standers, and my own knowledge of mischievous effects, related in books, I was not the least alarmed, and no accident happened.

It must be admitted, that deep rooted affections of the mind, fear, or anger, after a more or less distant period from the bite, are determining causes of hydrophobia. In the same way, after a wound in the neighbourhood of the nervous and membranous parts of the extremities, tetanus sometimes comes on from the following causes. 1st. A peculiar predisposition or susceptibility of the subjects. 2d. An increased sensibility and irritability. 3d. The nature of the air and of the place inhabited by the patient. And 4th. From the perspiration being checked. Might we not establish a kind of analogy between the symptoms occasioned by a bite, and the general spasm or tetanus?* It is certainly according to this apparent analogy, that Dr. Stutz of Suabia, proposes to apply his treatment of tetanus to hydrophobia. This method consists in administering fixed alkali alternately with opium, and in applying the warm bath saturated with this alkali, or rather with caustic potash.

When traumatic hydrophobia ensues from the above mentioned accidents, incisions and cautery are indispensable after the slightest appearance of the first symptoms, without

* It is with much justice that the Editors of the Journal de Medicine, last quoted, proposed to denominate *traumatic hydrophobia*, that which is occasioned by the bite of animals not rabid.

without neglecting opium afterwards internally, and oily embrocations.

I shall conclude this sketch with some curious experiments, made by inoculating with saliva. M. Huzard, in observations communicated to the Institute, says, that herbivorous animals do not seem to transmit hydrophobia by biting, as carnivorous animals do. In fact, we cannot communicate the disease by inoculating with the saliva of the former. Animals have also been inoculated without effect, with the saliva of persons who died of hydrophobia. But inoculation with the saliva of a mad dog almost always communicates this disease to other animals. Dr. Zinke made the following observations at Jena.

1. He dipt a camel's hair pencil in the saliva of a dog which had died rabid, after having bitten several other animals, which shared the same fate; he inoculated it in the fore legs of another dog on the same day; he covered and tied up the incisions, in order to hinder the animal from licking them, and he administered *belladonna*. On the eighth day he refused his food, became sad, and on the tenth day he was seized with rabies.

2. A second dog was inoculated in three places, with a mixture of the saliva with which the pencil was wet, and a strong solution of white arsenic in water. Two hours afterwards the bandages were removed, and the wounds were moistened with arsenical solution. On the third day the edges of the wound were inflamed and covered with crust, under which there was a little matter. It discovered no symptoms of hydrophobia.

3. A cat was inoculated with the same saliva, diluted in tincture of cantharides. The incisions were twice rubbed with ointment of cantharides. On the third day the cat refused its food, and sought to hide itself. On the ninth it became rabid and was killed.

4. The same physician inoculated a rabbit with a mixture of rabid saliva, and drops of volatile alkali. Four hours afterwards, he washed the wound with this alkali, and covered it with a cloth moistened with the same remedy. The animal became rabid on the eleventh day.

5. Another rabbit was inoculated with the saliva of a mad dog, diluted with the saliva of a healthy person. Two hours afterwards, the wounds were washed with soap ley. This bathing was repeated at the same intervals, and no hydrophobia ensued.

6. A dog was inoculated with the same saliva, diluted in a little water, in which some phosphorus had been put. Six

Six hours afterwards, the wounds were washed with phosphorated water. On the third day the wounds were crusted over and inflamed. On the fifth day the animal lost its appetite, but it did not become rabid.

7. A cock was inoculated with the same saliva, mixed with some of the gastric juice of a cat. Two hours afterwards, the wounds were rubbed with a small tooth brush, soaked in vinegar. One hour afterwards, they were rubbed with gastric liquor, and four hours afterwards with tincture of cantharides. Suppuration was kept up with tincture of cantharides, but on the fourteenth day the cock was seized with hydrophobia.

In five cases of bites of a mad dog, Dr. Zinke succeeded by rubbing the wounds with a small brush dipped in soap ley, and by making incisions. Afterwards he put the patients into a warm bath, then into a warm bed, and he gave hot drinks. When the bleeding had ceased, he covered it with a paste composed of white arsenic, like that which is generally applied to cancers, and he gave internally phosphorus dissolved in ether. This topical application produced only pain and inflammation, and yet it is at present regarded as a specific in the treatment of these bites.

The corollary to be drawn from the proposition I have placed at the beginning of my letter, consists therefore in the superiority of the local treatment, not only instantly after the bite, but also at any subsequent period.

I do not mean to say that the cautery should exclude the use of internal remedies, but without burning they are very rarely efficacious.

